# Depression, smoking, physical inactivity and season independently associated with midnight salivary cortisol in type 1 diabetes

**DOI:** 10.1186/1472-6823-14-75

**Published:** 2014-09-16

**Authors:** Eva O Melin, Maria Thunander, Mona Landin-Olsson, Magnus Hillman, Hans O Thulesius

**Affiliations:** 1 Department of Clinical Sciences, Endocrinology and Diabetes, Lund University, Lund, Sweden; 2 Primary Care, Kronoberg County Council, Växjö, Sweden; 3 Department of Research and Development, Kronoberg County Council, Växjö, Sweden; 4 Department of Internal Medicine, Central Hospital, Kronoberg County Council, Växjö, Sweden; 5 Department of Internal Medicine, Lund University Hospital, Lund, Sweden; 6 Department of Clinical Sciences, Family Medicine, Lund University, Malmö, Sweden; 7 Box 1223, 351 12 Växjö, Sweden

**Keywords:** Midnight salivary cortisol, Depression, Type 1 diabetes, Smoking, Physical activity, Season, HbA1c

## Abstract

**Background:**

Disturbances of the circadian rhythm of cortisol secretion are associated with depression, coronary calcification, and higher all-cause and cardiovascular mortality.

The primary aim of this study was to test the associations between midnight salivary cortisol (MSC), depression and HbA1c, and control for behavioural, environmental and intra individual factors with possible impact on cortisol secretion, like smoking, physical inactivity, season, medication, diabetes duration, severe hypoglycemia episodes, age and gender in patients with type 1 diabetes. Secondary aims were to present MSC levels for a reference group of non-depressed type 1 diabetes patients with a healthy life style (physically active and non-smoking), and to explore seasonal variations.

**Methods:**

A cross-sectional population based study of 196 patients (54% men and 46% women) aged 18–59 years that participated in a randomized controlled trial targeting depression in type 1 diabetes. Depression was assessed by the Hospital Anxiety and Depression Scale-depression subscale. MSC, HbA1c, serum-lipids, blood pressure, waist circumference and data from medical records and the Swedish National Diabetes Registry were collected.

**Results:**

Thirty four patients (17%) had MSC ≥9.3 nmol/L, which was associated with smoking (AOR 5.5), spring season (AOR 4.3), physical inactivity (AOR 3.9), self-reported depression (AOR 3.1), and older age (per year) (AOR 1.08). HbA1c >70 mmol/mol (>8.6%) (AOR 4.2) and MSC ≥9.3 nmol/L (AOR 4.4) were independently linked to self-reported depression. Season was strongly associated with MSC levels and no other variables studied showed seasonal variations. In a reference group of 137 non-depressed patients with a healthy life style (physically active, non-smoking) the median MSC level was 4.6 nmol/L (range 1.9–23.0).

**Conclusions:**

In this study of patients with type 1 diabetes high MSC was linked to smoking, physical inactivity, depression, season and older age. Thus a high cortisol value identified three major targets for treatment in type 1 diabetes.

## Background

Depression is common in persons with diabetes [1,2], affects women twice as often as men [3], and is associated with impaired glycemic control [1,2,4], diabetes complications [1,2], and all-cause mortality [5]. Hyperactivity of the hypothalamic-pituitary-adrenal (HPA) axis is found in both depression and in type 1 diabetes [2,3,6], though atypical depression is characterized by a down-regulated HPA axis [3]. In the extreme case of hypercortisolemia as in Cushing’s disease, 50–80% of the patients are depressed at the time of diagnosis [7]. Corticosteroids inhibit hippocampal serotonin receptors, and hypercortisolemia is linked to neurodegeneration and decreased hippocampus size, all important factors in depressive disorders and for cognitive function [2]. Improved HPA axis function and reduced cortisol levels are observed in patients with recovery from depression [2,6,8]. Apart from depression and cognitive impairment [9], hypercortisolemia is linked to abdominal obesity, sarcopenia, hypertension, diabetes, dyslipidemia, immunity changes, osteoporosis, arteriosclerosis and cardiovascular disease [3,7,10,11]. A disturbance of the circadian rhythm of cortisol, characterized by a flatter diurnal cortisol slope, is seen in depressed persons [12]. This type of disturbance is also associated with coronary calcification [13], and higher all-cause and cardiovascular mortality [14]. Seasonal variations are observed for cortisol secretion [15-17], depressive symptoms in seasonal affective disorder (SAD) [17], and in suicide incidence with a peak in spring in temperate climates [18]. Seasonal changes in depressive symptoms are considered to be the result of a failure to adapt to the shift in day length that accompanies seasonal change [19]. Light is the most important time-marker for entraining the circadian rhythms in physiology [20], and the hours of daylight in Sweden vary widely according to season. Higher salivary cortisol secretion is observed in women and in older persons [21]. Smoking is linked to high salivary cortisol excretion, high HbA1c and depressive symptoms [4,22,23]. Physical activity reduces depression, augments the benefits of antidepressant use [24,25], and physical fitness attenuates increased age related cortisol responses to stress [26]. Antidepressants are associated with alterations of the HPA axis [27]. Salivary cortisol follows the circadian rhythm with low levels at night and reflects the bioactive free molecule below plasma cortisol 500 nmol/L [28-33]. Salivary cortisol is increasingly used to assess hypercortisolism as sampling is noninvasive, painless and stress free [8,10,12-15,17,20,22,28-33].

The main hypothesis of this study was that a disturbed circadian rhythm manifested by high midnight cortisol is associated with depression and with impaired glycemic control in patients with type 1 diabetes. The primary aim of this study was to test the associations between midnight salivary cortisol (MSC), depression and HbA1c, and control for behavioural, environmental and intra individual factors with possible impact on cortisol secretion like smoking, physical inactivity, season, medication, diabetes duration, severe hypoglycemia episodes, age and gender in patients with type 1 diabetes. Secondary aims were to present MSC levels for a reference group of non-depressed type 1 diabetes patients with a healthy life style (physically active and non-smoking), and to explore seasonal variations.

## Methods

This study presents cross sectional baseline data from the randomized control trial (RCT) “Psychological variables and hyperglycemia in diabetes mellitus” (ClinicalTrials.gov: NCT01714986) which targets psychological symptoms in patients with diabetes and inadequate glycemic control in a population based cohort of patients with type 1 diabetes. A first baseline study showed that depression, obesity and smoking were independently associated with high HbA1c [4]. Results of the intervention arms “Affect School with Script Analysis” and “Basic Body Awareness Therapy” [34,35] will be followed up in 2015 with primary outcome prevalence of depression and secondary outcomes HbA1c levels and prevalence of alexithymia and anxiety.

### Participants and procedures

To explore variables associated with high MSC we consecutively recruited 196 persons. Patients attended the only specialist diabetes outpatient clinic in a county with a population of 125,000 in South Sweden during 2009 [4]. Inclusion criteria were age 18–59 years and type 1 diabetes duration for at least 1 year. Exclusion criteria were pregnancy, severe somatic comorbidities or diabetes complications (cancer, hepatic failure, or end-stage renal disease), severe mental disorders (psychotic disorder, bipolar disorder, severe personality disorder, severe substance abuse, mental retardation, or other severe cognitive deficiencies), systemic corticosteroid treatment, visual impairment to such a degree that reading the questionnaires was impossible, or inadequate knowledge of Swedish. Two patients with eczema and psoriasis, both non-smokers, non-depressed, physically active, and normotensive, had very high MSC (82 and 72 nmol/L) and were excluded since topical steroid contamination was suspected.

There were 62 patients who chose not to deliver MSC samples and 23 who failed to deliver proper samples. These 85 patients did not differ from the 196 included patients regarding smoking (p = 0.13), age (p = 0.15), gender (p = 0.30), use of antidepressants (p *=* 0.33), mean HbA1c (p = 0.34), abdominal obesity (p = 0.38), physical inactivity (p = 0.68), clinical psychiatric diagnosis (p *=* 0.71), hypertension (p = 0.80), hyperlipidemia (p = 0.80), diabetes duration (p = 0.89), or self-reported depression (p >0.99).

The 196 patients underwent self-reported depression assessment and their MSC, HbA1c, serum-lipids, waist circumference (WC) and blood pressure were measured.

Data were also collected from the Swedish National Diabetes Register (S-NDR), and from computerized medical records from the Departments of Internal Medicine, Ophthalmology, and Psychiatry (only drug prescription data), and from Primary Care clinics.

The study was approved by the Regional Ethical Review Board of Linköping University (Registration no. M120-07, T89-08). All patients provided written informed consent.

### Midnight salivary cortisol (MSC)

Each patient collected one MSC sample between 23.30 and 00.30 hours, using the Salivette sampling method (Salivette®, Sarstedt, Nümbrecht, Germany) [8,13,22,29-33]. Patients had a restriction period of 30 minutes prior to sampling when they were told not to eat, drink, smoke, use snuff, or perform physical exercise [20,31,32], and avoid brushing their teeth 60 minutes before sampling. They were instructed to put the swab below the tongue until wet, store the sample in a refrigerator, and mail it to the laboratory the next morning. The samples where centrifuged and frozen at -25 centigrades until assayed at the Department of Clinical Chemistry, Lund University Hospital, Lund. The Roche Cobas Cortisolassay®, a competitive Electrochemiluminescence immunoassay (ECLIA) was used on an Elecsys 2010 immunoanalyser system (Roche Diagnostics, Mannheim, Germany) [29-32].

In a healthy population without diabetes, late night cortisol ranged from 1.4 to 16.7 nmol/L, and the 95^th^ percentile was 8.9 nmol/L [30]. Mean MSC (analysed with Salivary Cortisol ELISA SLV-2930) for persons with pseudo-Cushing’s syndrome was 7.7 ± 1.0 nmol/l [8]. To distinguish Cushing’s disease from pseudo-Cushing’s syndrome a cut-off value of MSC ≥9.3 nmol/L was suggested in the same study, corresponding to a sensitivity of 100% and a specificity of 83% [8]. Therefore MSC ≥9.3 nmol/L was defined as a high MSC level with clinical significance in this study.

### Season, self-reported depression, clinical psychiatric diagnoses and life-style

MSC samples were collected between 29/03/2009 and 18/01/2010, which was divided into three periods. The first period 29/03/2009 until 31/05/2009 was defined as spring; the second period 01/06/2009 until 31/08/2009 was defined as summer; and the third period 09/01/2009 until 18/01/2010 was defined as autumn/winter.

Self-reported depression was assessed by Hospital Anxiety and Depression Scale-depression subscale (HADS-D) consisting of 7 statements with 4 response alternatives from 0 to 3, using the recommended ≥8 points as cut off level [4,9,34,36]. Positive associations between self-reported depression and clinical psychiatric diagnosis with and without use of antidepressants confirmed the validity of the HADS-D.

Clinical psychiatric diagnoses were established clinically prior to recruitment, were dichotomized as having or not having a psychiatric diagnosis, and used mainly for validation of the HADS-D.

Smokers were defined as patients having smoked any amount of tobacco during the last year.

Physical inactivity was defined as moderate activities, such as 30 minutes of walking, less than once a week.

### Metabolic variables and hypoglycemia episodes

Venous HbA1c was analysed with high pressure liquid chromatography, HPLC - variant II, Turbo analyzer (Bio – Rad®, Hercules, CA, USA) [37]. HbA1c was converted from Mono-S and dichotomized at the third quartile (q_3_)_,_ which was defined as high HbA1c (Mono-S >7.7%, DCCT >8.6%, IFCC >70 mmol/mol) [38].

Serum-lipids were analysed with the enzymatic colour test (Olympus AU®, Tokyo, Japan). Hyperlipidemia was defined as S-Cholesterol >4.5 mmol/L and/or S-Low density lipoprotein cholesterol >2.5 mmol/L (according to the Swedish national guidelines for diabetes management); or use of lipid lowering drugs independent of lipid blood levels. HbA1c and lipids were analysed at the department of Clinical Chemistry, Växjö Central Hospital.

Blood pressure was measured in the sitting position. Hypertension was defined as systolic blood pressure >130 mm Hg and/or diastolic blood pressure >80 mm Hg (according to the Swedish national guidelines for diabetes management); or use of antihypertensive drugs independent of blood pressure levels.

WC was measured between the lowest rib margin and iliac crest by a nurse. Abdominal obesity was defined as WC ≥1.02 m. for men, and as WC ≥0.88 m. for women [4].

A severe hypoglycemic episode was defined as needing help from another person due to hypoglycemia, and episodes occurring during the last 6 months were registered [4].

### Statistical analysis

SPSS® version 18 (IBM, Chicago, Illinois, USA) was used for statistical analyses. Fisher’s exact test (two-tailed) was used to analyse differences of prevalence. Continuous variables, normally distributed, were presented as mean ± SD, and Student’s *t*-test was used for analyses of mean differences. Non-parametric distribution was presented as median values (quartile (q)_1,_ q_3_; range), and analyses were performed with Kruskal-Wallis test or Mann-Whitney *U* test. MSC was dichotomized at 9.3 nmol/L [8], and HbA1c at 70 mmol/mol (8.6%) [4]. Crude odds ratios (CORs) were calculated. Variables with p ≤0.20 and gender were entered into multiple logistic regression analysis (Backward: Wald) with MSC ≥9.3 nmol/L as dependent variable. Life style variables, antidepressants, season, age, gender, MSC ≥9.3 nmol/L and HbA1c >70 mmol/L (>8.6%), were entered into multiple logistic regression analysis with self-reported depression as dependent variable. Confidence intervals (CIs) of 95% were used. *P* ≤0.05 was considered statistically significant.

## Results

In this study of 196 patients with type 1 diabetes, 54% men and 46% women with mean age 41.3 (range 18–59) years and mean diabetes duration 21.1 (range 1–55) years, we analyzed variables associated with high MSC levels and self-reported depression. Baseline characteristics and gender differences are presented in Table 1. Twenty one (11%) patients used continuous subcutaneous insulin infusion and 175 (89%) used multiple daily insulin injections. There were 137 (70%) non-depressed (self-reported), non-smoking and physically active patients; 45 (23%) patients were either depressed, smokers or physically inactive, or had combinations of these variables; and 14 (7%) were non-depressed but with missing data regarding life style factors. Clinical psychiatric diagnoses were established in 27 (14%) patients and 13 used antidepressants. Their clinical diagnoses were depression (n = 16), anxiety disorder (n = 4), stress related disorder (n = 4), controlled alcohol addiction (n = 2), or attention deficit hyperactivity disorder (n = 1).

**Table 1:** Baseline characteristics and gender differences in 196 patients with type 1 diabetes

		**All patients (n = 196)**	**Men (n = 106)**	**Women (n = 90)**	**P**^**1**^
Age (years)		41.3 ± 11.7	42.6 ± 12.0	39.7 ± 11.2	0.083^2^
Diabetes duration (years)		21.1 ± 12.2	22.3 ± 12.5	19.7 ± 11.8	0.14^2^
High MSC					
MSC ≥9.3 nmol/L		34 (17)	17 (16)	17 (19)	0.71
Psychiatric variables					
Depression^3^		20 (10)	12 (11)	8 (9)	0.64
Clinical psychiatric diagnoses		27 (14)	8 (8)	19 (21)	0.007
Life style factors					
Smoking^4^		16 (9)	11 (11)	5 (6)	0.30
Physical inactivity^5^		19 (10)	10 (10)	9 (11)	> 0.99
Metabolic variables and hypoglycemia					
HbA1c	mmol/mol	62 ± 13	62 ± 10	64 ± 15	0.30^2^
	%	7.9 ± 1.1	7.8 ± 1.0	8.0 ± 1.3	
HbA1c >70 mmol/mol (>8.6%)		50 (26)	22 (21)	28 (31)	0.10
Abdominal obesity^6^		29 (15)	8 (8)	21 (24)	0.002
Hypertension		106 (54)	65 (61)	41 (46)	0.031
Hyperlipidemia		167 (85)	96 (91)	71 (79)	0.027
Severe hypoglycemia episodes^7^		9 (5)	4 (4)	5 (5)	0.74
Medication					
Antidepressants		13 (7)	4 (4)	9 (10)	0.092
Antihypertensive medication		60 (31)	38 (36)	22 (24)	0.090
Lipid lowering drugs		93 (47)	53 (50)	40 (44)	0.48
Inhaled steroids		15 (8%)	4 (4)	11 (12)	0.032

Data are means ± SD or *n* (%). ^1^Fisher’s exact test unless otherwise indicated. ^2^Student’s *t*-test. ^3^Self-reported. ^4^Smoking: 10 missing values. ^5^Physical inactivity: 12 missing values. ^6^WC: men ≥1.02 m; women ≥0.88 m. ^7^At least one severe hypoglycemia episode during the last 6 months where they needed help from another person.

### MSC for all patients included in the study

For all 196 patients median MSC was 5.0 (q_1_, q_3_, range: 3.1, 7.5; 1.9–47.0) nmol/L (Table 2). Median MSC levels were higher for patients that were smokers (p <0.001), had self-reported depression (p = 0.005), or were physically inactive (p = 0.050) (Table 2). Median MSC did not differ between users and non-users of antidepressants in patients with self-reported depression (p = 0.76), and not in patients without self-reported depression (p = 0.90) (Table 2).

**Table 2:** Midnight salivary cortisol (MSC) by gender, psychiatric factors, lifestyle, obesity, high HbA1c, hypoglycemia, and medication in 196 patients with type 1 diabetes

	**Midnight salivary cortisol (nmol/L)**			
		**n (%)**	**Median (q**_**1**_**, q**_**3; **_**range)**	**P**^**1**^
All participants		196	5.0 (3.1, 7.5; 1.9–47.0)	
Gender				
Men		106 (54)	4.6 (3.1, 6.8; 1.9–47.0)	0.062
Women		90 (46)	5.6 (3.2, 8.0; 1.9–23.0 )	
Psychiatric variables				
Depression^2^	Yes	20 (10)	7.7 (5.0, 13.0; 1.9–31.0)	0.005
	No	176 (90)	4.8 (3.0, 7.1; 1.9–47.0)	
Depression^2^ and antidepressants (Sub analysis)				
Depression^2^, using antidepressants		5 (2)	8.7 (3.3; 18.0; 3.0–26.0)	0.76
Depression^2^, not using antidepressants		15 (8)	6.7 (5.1; 13.0; 1.9–31.0)	
No depression^2^, using antidepressants		8 (4)	4.4 (3.1; 8.6; 2.9–14.0)	0.90
No depression^2^, not using antidepressants		168 (86)	4.8 (3.0; 7.1; 1.9–47.0)	
Clinical psychiatric diagnoses	Yes	27 (14)	5.3 (3.7, 9.4; 1.9–26.0)	0.28
	No	169 (86)	5.0 (3.0, 7.4; 1.9–47.0)	
Life style factors				
Smoking	Yes	16 (9)	9.0 (6.6, 11.8; 2.3–47.0)	<0.001
	No	170 (91)	4.8 (3.0, 7.0; 1.9–31.0)	
Physical inactivity	Yes	19 (10)	6.3 (4.3, 13.0; 1.9–31.0)	0.050
	No	165 (90)	4.9 (3.0, 7.2; 1.9–47.0)	
Metabolic variables and hypoglycemia episodes				
HbA1c >70 mmol/mol (>8.6%)	Yes	50 (26)	5.3 (3.7, 7.6; 1.9–31.0)	0.26
	No	146 (74)	4.8 (3.0, 7.5; 1.9–47.0)	
Abdominal obesity, men^3^	Yes	8 (8)	3.8 (2.5, 5.4; 1.9–31.0)	0.37
	No	96 (92)	4.8 (3.1, 7.2; 1.9–47.0)	
Abdominal obesity, women^4^	Yes	21 (24)	7.1 (5.1, 8.8; 2.9–20)	0.030
	No	65 (76)	5.0 (2.9, 7.8; 1.9–23.0)	
Severe hypoglycemia episodes^5^	Yes	9 (5)	5.4 (3.4, 6.5; 2.4–11.0)	0.96
	No	186 (95)	5.0 (3.1, 7.6; 1.9–47.0)	
Medication				
Antidepressants	Yes	13	4.4 (3.3, 9.7; 2.9–26.0)	0.53
	No	183	5.0 (3.1, 7.4; 1.9–47.0)	
Inhaled steroids	Yes	15 (8)	5.4 (3.0, 7.6; 2.3–11.0)	0.88
	No	181 (92)	5.0 (3.1, 7.5; 1.9–47.0)	

^1^Mann-Whitney *U* test. ^2^Self-reported. ^3^WC: ≥1.02 m. ^4^WC: ≥0.88 m. ^5^At least one severe hypoglycemia episode during the last 6 months where they needed help from another person.

Median MSC levels (p <0.001) and the prevalence rates of high MSC (≥9.3 nmol/L) (p = 0.013) were highest in the spring samples and lowest in the autumn/winter samples (Table 3). No seasonal clustering was observed for physical inactivity, smoking, self-reported depression, HbA1c, gender or age (Table 3).

**Table 3:** Exploration of seasonal clustering in 196 patients with type 1 diabetes

		**Seasons**			
		**Spring**^**1 **^**(n = 79)**	**Summer**^**2 **^**(n = 50)**	**Autumn/winter**^**3 **^**(n = 67)**	**P**^**4**^
MSC	nmol/L	6.7 (4.7, 9.3)	4.6 (2.8, 6.8)	3.5 (2.7, 5.5)	<0.001^5^
MSC ≥9.3 nmol/L	Yes	20 (25)	9 (18)	5 (8)	0.013
	No	59 (75)	41 (82)	62 (92)	
Age (years)		45.0 (32.0, 52.0)	40.0 (28.0, 48.2)	44.0 (32.0, 53.0)	0.090^5^
Physical inactivity^6^	Yes	9 (12)	7 (15)	3 (5)	0.21
	No	66 (88)	41 (85)	58 (95)	
Gender	Men	39 (49)	32 (64)	35 (49)	0.25
	Women	40 (51)	18 (36)	32 (51)	
High HbA1c^7^	Yes	23 (29)	15 (30)	12 (18)	0.21
	No	56 (71)	35 (70)	55 (82)	
HbA1c	mmol/mol	63 (53, 71)	64 (55, 72)	60 (53, 68)	0.32^5^
	%	7.9 (7.0, 8.6)	8.0 (7.2, 8.8)	7.7 (7.0, 8.4)	
Smoking^8^	Yes	9 (12)	4 (8)	3 (5)	0.32
	No	66 (88)	45 (92)	59 (95)	
Depression^9^	Yes	11 (14)	4 (8)	5 (8)	0.41
	No	68 (86)	46 (92)	62 (92)	

Data are n (%) or median (q_1_, q_3_). ^1^(29/03/2009–31/05/2009). ^2^(01/06/2009–31/08/2009). ^3^(01/09/2009–18/01/2010). ^4^Fisher’s exact test unless otherwise indicated. ^5^Kruskal-Wallis test. ^6^Physical inactivity: 12 missing values. ^7^HbA1c >70 mmol/mol (>8.6%). ^8^Smoking: 10 missing values. ^9^Self-reported.

Thirty four patients (17%) had MSC ≥9.3 nmol/L, which was associated with smoking (AOR 5.5), spring (AOR 4.3), physical inactivity (AOR 3.9), self-reported depression (AOR 3.1), and older age (per year) (AOR 1.08) (Table 4).

**Table 4:** Associations with high midnight salivary cortisol (MSC) for 181 patients with type 1 diabetes

		**High midnight salivary cortisol (≥9.3 nmol/L)**			
		**COR (95% CI)**	**P**^**1**^	**AOR (95% CI)**	**P**^**2**^
Smoking		5.5 (1.9–16.1)	0.002	5.5 (1.6–18.5)	0.006
Age (per year)		1.06 (1.02–1.10)	0.002	1.08 (1.03–1.13)	0.001
Season					
Spring		4.2 (1.5–11.9)	0.007	4.3 (1.4–13.7)	0.013
Summer		2.7 (0.9–8.7)	0.09	3.4 (0.9–13.0)	0.07
Autumn/winter (reference)		1		1	
Physical inactivity		3.0 (1.1–8.3)	0.036	3.9 (1.1–13.4)	0.032
Depression		4.9 (1.9–13.1)	0.001	3.1 (1.0–9.2)	0.047
Women		1.2 (0.6–2.6)	0.66	2.2 (0.9–5.2)	0.089
Antidepressants		2.3 (0.7–7.8)	0.20	-	0.76
Inhaled steroids		0.3 (0.04–2.5)	0.28	-	-
Diabetes duration		1.01 (0.98–1.04)	0.40	-	-
HbA1c	mmol/mol (per unit)	1.01 (0.98–1.04)	0.57	-	-
	% (per unit)	1.10 (0.80–1.51)			
Abdominal obesity, men		0.7 (0.1–6.2)	0.76	-	-
Abdominal obesity, women		0.9 (0.3–3.3)	0.92	-	-

Missing lifestyle variables for 15 persons (smoking and/or physical inactivity). ^1^Simple logistic regression. ^2^Multiple logistic regression analysis (Backward: Wald). Nagelkerke R Square = 0.311.

### MSC in non-depressed (self-reported), non-smoking and physically active patients

Median MSC (q_1_, q_3_; range; 5^th^ percentile; 95^th^ percentile) nmol/L was 4.6 (3.0, 6.8; 1.9–23.0; 2.0; 12.0) for 137 (55% men) non-depressed, non-smoking and physically active patients with median (range) age 43 (20–59) years. In these 137 patients MSC ≥9.3 nmol/L was associated with season, spring (AOR 7.9 (1.6–37.8), p = 0.010), summer (AOR 1.9 (0.2–14.5), p = 0.54), autumn/winter (AOR 1) (reference); but not with age (per year) (1.05 (0.99–1.11), p = 0.084), or gender (p = 0.59). In spring median MSC (q_1_, q_3_; range) nmol/L was (n = 50) 6.6 (4.5, 8.8; 1.9–23.0), in summer (n = 36) 3.5 (2.7, 5.3; 1.9–14.0); and in autumn/winter (n = 51) 3.5 (2.8, 5.4; 1.9–11.0), p <0.001. Median age (range) years in spring was 46 (20–59); in summer 39 (20–59); in autumn/winter 44 (22–59), p = 0.022. Median age did not differ between patients recruited in spring and autumn/winter (p = 0.67).

### Associations with self-reported depression

MSC ≥9.3 nmol/L (AOR 4.4), HbA1c >70 mmol/L (>8.6%) (AOR 4.2) and antidepressants (AOR 4.9) were independently associated with self-reported depression (Table 5).

**Table 5:** Associations with self-reported depression for 181 patients with type 1 diabetes

	**Self-reported depression**			
	**COR (95% CI)**	**P**^**1**^	**AOR (95% CI)**	**P**^**2**^
MSC ≥9.3 nmol/L	4.9 (1.9–13.1)	0.001	4.4 (1.5–13.0)	0.007
HbA1c >70 mmol/L (>8.6%)	4.3 (1.7–11.1)	0.003	4.2 (1.5–11.8)	0.007
Antidepressants	7.0 (2.0–24.1)	0.002	4.9 (1.2–20.8)	0.030
Women	0.8 (0.3–2.0)	0.58	-	0.17
Physical inactivity^3^	3.6 (1.1–11.3)	0.030	-	0.18
Age (per year)	1.04 (1.0–1.1)	0.060	-	0.29
Season				
Spring	2.0 (0.7–6.1)	0.22	-	0.33
Summer	1.1 (0.3–4.2)	0.91	-	0.33
Autumn/winter (reference)	1		1	
Smoking^3^	2.1 (0.5–8.0)	0.29	-	0.68
Clinical psychiatric diagnosis	7.2 (2.6–19.7)	<0.001	-	-

Missing life style values for 15 persons. ^1^Simple logistic regression. ^2^Multiple logistic regression analysis (Backward: Wald). Nagelkerke R Square = 0.23.

### Validation of the HADS-D

The associations (COR (CI), p) were significant between self-reported depression and “clinical psychiatric diagnosis and use of antidepressants” (9.0 (2.5–32.1), 0.001 (n = 13)), and between self-reported depression and “clinical psychiatric diagnosis without use of antidepressants” (5.7 (1.5–21.3), 0.009 (n = 14)), with “no clinical psychiatric diagnosis/antidepressants” as reference (n = 169).

## Discussion

**Figure 1:**
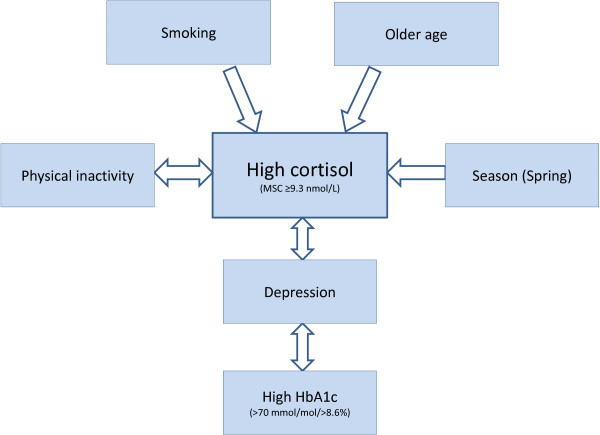
**Links with high MSC (≥9.3 nmol/L) and depression.** Depression, smoking, physical inactivity, age and season (spring) were linked to high MSC. High HbA1c and high MSC were independently linked to depression. The arrows are drawn bidirectional between all variables where bidirectional links can’t be excluded: between depression and high HbA1c, between depression and high MSC, and finally between physical inactivity and high MSC.

In this population based study of 196 patients with type 1 diabetes, smoking, physical inactivity, depression and age, were associated with high MSC (≥9.3 nmol/L), whereas HbA1c was not. High MSC and high HbA1c (>70 mmol/L (>8.6%)) were independently associated with depression. A seasonal variation was found with the highest prevalence of high MSC levels in spring and the lowest in autumn/winter. The main links between these variables are illustrated in Figure 1.

Strengths of our study are first that we systematically investigated factors that could confound our results such as use of antidepressants or inhaled steroids, and seasonal changes in cortisol secretion. Secondly, we determined factors not associated with high MSC, i.e. HbA1c, antidepressants and diabetes duration. Third, the population of patients with type 1 diabetes was large and well defined. Pregnant women, patients with severe somatic or psychiatric disorders including substance abuse, and patients using systemic corticosteroid treatment, were excluded, all factors that knowingly affect cortisol levels. Fourth, we thoroughly examined and found that the eligible 85 patients who did not deliver salivary cortisol samples did not differ from the 196 included patients. This suggests that our results could be generalized to a larger population of patients with type 1 diabetes.

Limitations to our study are first that self-reported depression was not confirmed by a diagnostic interview. Yet, clinical psychiatric diagnoses, both for those using and not using antidepressants, were clearly associated with self-reported depression. Secondly, HbA1c and MSC were only measured once, but a demand for repeated measurements would probably have resulted in a lower participation rate due to the inconvenience with both venous and midnight sampling. Third, there was no data from the middle of January until the end of March which makes it impossible to exclude seasonality in depressive symptoms, though we did not find any. Fourth, to confirm the seasonality of MSC levels there is a need for repeated measurements throughout the year.

A normal circadian rhythm of cortisol is characterized by maximum levels in the morning and minimum levels at midnight [8]. In this study we chose to use MSC ≥9.3 nmol/L as cut-off, a very high level that was recently used to differentiate pseudo-Cushing’s syndrome from true Cushing’s disease [8]. The association between this very high level of midnight cortisol and self-reported depression indicates a disturbance of the circadian rhythm in depressed patients with type 1 diabetes. Depression has previously been linked to hyperactivity of the HPA axis [2,3], a disturbance of the circadian rhythm characterized by a flatter diurnal slope of cortisol secretion [12], a down regulated HPA axis in atypical depression [3], and seasonal variations with an attenuated cortisol awakening response in SAD during winter months [17].

We found the highest midnight cortisol levels in spring and the lowest in autumn/winter which is a new finding. First, we have not found any previous study where seasonal variations of midnight salivary cortisol were analysed; measurements have been performed during daytime and late evening. Second, according to a review of circannual hormonal changes, basal levels of circulating glucocorticoids seem to be lower during the spring and summer and peak during the autumn and winter [16]. However, one research group reported the highest cortisol concentrations at daytime in February, March, and April, (the high levels in March and April are findings quite consistent with ours), and the lowest concentrations in July and August (which differ from our findings) [15]. Another research group found that the cortisol awakening response was attenuated in persons with SAD during winter months but did not find any seasonal variations of cortisol secretion in healthy individuals [17]. Spring is in Sweden characterized by rapidly increasing light intensity and longer day light periods, which might influence cortisol secretion as light is an important time-marker for cortisol secretion [20]. The seasonality in MSC secretion was not explained by seasonal differences in self-reported depression, smoking or physical inactivity, or by uneven distribution of women/men or older/younger during the different seasons. Actually the association between high MSC and spring was very high (AOR 7.9) compared to autumn/winter in the non-depressed population with a healthy life style. Our results suggest that seasonal variations of MSC should be considered both when MSC is measured for clinical purposes, and in future research.

Hypercortisolemia is a known cause of hyperglycemia [7], but we found no direct association between MSC and HbA1c. The reason could be that the influence of hypercortisolemia on glycemic control was successfully counteracted by higher insulin doses; unfortunately we have no information of their insulin doses. Instead we found that depression was independently associated both with high MSC and with high HbA1c.

The absence of associations between depression and gender, physical inactivity and smoking differ from previous research [3,23,24]. Findings in our study of the links between high cortisol and smoking and older age are consistent with previous studies of people without diabetes [22,26].

We chose a 30 minutes (60 minutes for brushing the teeth) restriction period of eating etc. before MSC sampling. A variety of restriction periods before salivary cortisol sampling are found in the literature: 15 minutes [14,22], 30 minutes [20,31,32], 2 hours [8], and 3 hours [28]. How much a shorter or longer restriction period would affect the results is difficult to say, but a long restriction period might negatively affect the participation rate, and for patients with type 1 diabetes it is preferable not to interfere with ordinary mealtimes in order to avoid hypoglycemia episodes.

The ECLIA method used to analyze MSC in our study is well validated [29-32], but there are no established reference ranges for patients with type 1 diabetes. To aid future research and clinical assessments, reference MSC values were calculated for non-depressed, non-smoking physically active patients with type 1 diabetes, and reference ranges were also presented for the different seasons.

Salivary cortisol will probably be used more in future clinical practice and research as it can be sampled at home, is noninvasive, painless and stress free [29,32]. High levels of MSC particularly in younger non-smoking patients could indicate depression. Normalized cortisol levels have been observed after resolution of depressive symptoms [2,6,8], but if recovery from depressive symptoms will lead to decreased MSC levels in patients with type 1 diabetes is a subject for future research. Other subjects for future research are to explore and compare the effects on the HPA axis of the different subtypes of antidepressants, and to explore the effects of psycho education and stress reducing techniques on depression and cortisol secretion in patients with type 1 diabetes [34,35].

## Conclusions

High levels of MSC linked to depression, smoking and physical inactivity highlights three main targets in diabetes care, as a disturbance of the circadian rhythm of cortisol is associated with coronary calcification, all-cause and cardiovascular mortality [13,14].

The additional link between depression and high HbA1c emphasizes the severity of depression in patients with type 1 diabetes. Routine systematic depression evaluation at diabetes control visits is suggested. A high cortisol level may help to emphasize to medical professionals and patients alike the necessity of taking action against depression, smoking and physical inactivity.

## Abbreviations

AOR: Adjusted odds ratio; CI: Confidence interval; COR: Crude odds ratio; DCCT: Diabetes control and complication trial; ECLIA: Electrochemiluminescence immunoassay; HADS-D: Hospital anxiety and depression scale-depression subscale; HPA axis: Hypothalamic-pituitary-adrenal axis; IFCC: International federation of clinical chemistry; MSC: Midnight salivary cortisol; q_1_: The first quartile; q_3_: The third quartile; RCT: Randomized controlled trial; SAD: Seasonal affective disorder; S-NDR: Swedish national diabetes registry; WC: Waist circumference.

## Competing interests

The authors declare that they have no conflicts of interest that could be perceived as prejudicing the impartiality of the research reported.

## Authors’ contributions

EOM, MT, MH, ML-O and HOT participated as investigators and reviewed and edited the manuscript. EOM, ML-O, MT and MH contributed to the study design and implementation. EOM, HOT and ML-O contributed to the analysis and wrote the statistical methods. EOM wrote the manuscript, and is the guarantor of this work and, as such, had full access to all the data in the study and takes responsibility for the integrity of the data and the accuracy of the data analysis. All authors read and approved the final manuscript.

## Pre-publication history

The pre-publication history for this paper can be accessed here:

http://www.biomedcentral.com/1472-6823/14/75/prepub

## References

[B1] Anderson R Freedland K Clouse R Lustman P The prevalence of comorbid depression in adults with diabetes: a meta-analysis Diabetes Care 2001 24 1069 1078 10.2337/diacare.24.6.1069 11375373

[B2] Korczak DJ Pereira S Koulajian K Matejcek A Giacca A Type 1 diabetes mellitus and major depressive disorder: evidence for a biological link Diabetologia 2011 54 2483 2493 10.1007/s00125-011-2240-3 21789690

[B3] Gold PW Chrousos GP Organization of the stress system and its dysregulation in melancholic and atypical depression: high vs low CRH/NE states Mol Psychiatry 2002 7 254 275 10.1038/sj.mp.4001032 11920153

[B4] Melin EO Thunander M Svensson R Landin-Olsson M Thulesius HO Depression, obesity and smoking were independently associated with inadequate glycemic control in patients with type 1 diabetes Eur J Endocrinol 2013 168 861 869 10.1530/EJE-13-0137 23536618

[B5] Egede LE Nietert PJ Zheng D Depression and all-cause and coronary heart disease mortality among adults with and without diabetes Diabetes Care 2005 28 1339 1345 10.2337/diacare.28.6.1339 15920049

[B6] Gillespie CF Nemeroff CB Hypercortisolemia and depression Psychosom Med 2005 67 Suppl 1 26 28 10.1097/01.psy.0000163456.22154.d2 15953796

[B7] Feelders RA Pulgar SJ Kempel A Pereira AM The burden of Cushing’s disease: clinical and health-related quality of life aspects Eur J Endocrinol 2012 167 311 326 10.1530/EJE-11-1095 22728347

[B8] Alwani RA Schmit Jongbloed LW de Jong FH van der Lely AJ de Herder WW Feelders RA Differentiating between Cushing’s disease and pseudo-Cushing’s syndrome: comparison of four tests Eur J Endocrinol 2014 170 477 486 10.1530/EJE-13-0702 24394725

[B9] Reynolds RM Strachan MWJ Labad J Lee AJ Frier BM Fowkes FG Mitchell R Seckl JR Deary IJ Walker BR Price JF Investigators obotETDS Morning cortisol levels and cognitive abilities in people with type 2 diabetes: the Edinburgh type 2 diabetes study Diabetes Care 2010 33 714 720 10.2337/dc09-1796 20097784 PMC2845011

[B10] Dekker MJ Koper JW van Aken MO Pols HAP Hofman A de Jong FH Kirschbaum C Witteman JCM Lamberts SWJ Tiemeier H Salivary cortisol is related to atherosclerosis of carotid arteries J Clin Endocrinol Metab 2008 93 3741 3747 10.1210/jc.2008-0496 18682510

[B11] Reynolds RM Labad J Strachan MWJ Braun A Fowkes FGR Lee AJ Frier BM Seckl JR Walker BR Price JF Investigators obotETDS Elevated fasting plasma cortisol is associated with ischemic heart disease and its risk factors in people with type 2 diabetes: the Edinburgh type 2 diabetes study J Clin Endocrinol Metab 2010 95 1602 1608 10.1210/jc.2009-2112 20130072 PMC3971455

[B12] Knight JM Avery EF Janssen I Powell LH Cortisol and depressive symptoms in a population-based cohort of midlife women Psychosom Med 2010 72 855 861 10.1097/PSY.0b013e3181f4ab87 20841562 PMC3115732

[B13] Matthews K Schwartz J Cohen S Seeman T Diurnal cortisol decline is related to coronary calcification: CARDIA study Psychosom Med 2006 68 657 661 10.1097/01.psy.0000244071.42939.0e 17012518

[B14] Kumari M Shipley M Stafford M Kivimaki M Association of diurnal patterns in salivary cortisol with all-cause and cardiovascular mortality: findings from the Whitehall II study J Clin Endocrinol Metab 2011 96 1478 1485 10.1210/jc.2010-2137 21346074 PMC3085201

[B15] Persson R Garde AH Hansen AM Osterberg K Larsson B Orbaek P Karlson B Seasonal variation in human salivary cortisol concentration Chronobiol Int 2008 25 923 937 10.1080/07420520802553648 19005896

[B16] Cahill S Tuplin E Holahan MR Circannual changes in stress and feeding hormones and their effect on food-seeking behaviors Front Neurosci 2013 7 140 23966906 10.3389/fnins.2013.00140 PMC3735984

[B17] Thorn L Evans P Cannon A Hucklebridge F Clow A Seasonal differences in the diurnal pattern of cortisol secretion in healthy participants and those with self-assessed seasonal affective disorder Psychoneuroendocrinology 2011 36 816 823 10.1016/j.psyneuen.2010.11.003 21145663

[B18] Postolache TT Mortensen PB Tonelli LH Jiao X Frangakis C Soriano JJ Qin P Seasonal spring peaks of suicide in victims with and without prior history of hospitalization for mood disorders J Affect Disord 2010 121 88 93 10.1016/j.jad.2009.05.015 19535151 PMC2837087

[B19] Boyce P Barriball E Circadian rhythms and depression Aust Fam Physician 2010 39 307 310 20485718

[B20] Scheer F Buijs R Light affects morning salivary cortisol in humans J Clin Endocrinol Metab 1999 84 3395 3398 10.1210/jcem.84.9.6102 10487717

[B21] Larsson CA Gullberg B Rastam L Lindblad U Salivary cortisol differs with age and sex and shows inverse associations with WHR in Swedish women: a cross-sectional study BMC Endocr Disord 2009 9 16 10.1186/1472-6823-9-16 19545400 PMC2711063

[B22] Badrick E Kirschbaum C Kumari M The relationship between smoking status and cortisol secretion J Clin Endocrinol Metab 2007 92 819 824 10.1210/jc.2006-2155 17179195

[B23] Berlin I Covey LS Glassman AH Smoking and depression: a co-morbidity J Dual Diagn 2009 5 149 158 10.1080/15504260902870129

[B24] Dunn AL Trivedi MH Kampert JB Clark CG Chambliss HO Exercise treatment for depression: efficacy and dose response Am J Prev Med 2005 28 1 8 15626549 10.1016/j.amepre.2004.09.003

[B25] Hoffman BM Babyak MA Craighead WE Sherwood A Doraiswamy PM Coons MJ Blumenthal JA Exercise and pharmacotherapy in patients with major depression: one-year follow-up of the SMILE study Psychosom Med 2011 73 127 133 10.1097/PSY.0b013e31820433a5 21148807 PMC3671874

[B26] Traustadottir T Bosch PR Matt KS The HPA axis response to stress in women: effects of aging and fitness Psychoneuroendocrinology 2005 30 392 402 10.1016/j.psyneuen.2004.11.002 15694119

[B27] Manthey L Leeds C Giltay EJ van Veen T Vreeburg SA Penninx BWJH Zitman FG Antidepressant use and salivary cortisol in depressive and anxiety disorders Eur Neuropsychopharmacol 2011 21 691 699 10.1016/j.euroneuro.2011.03.002 21458959

[B28] Putignano P Toja P Dubini A Pecori Giraldi F Corsello SM Cavagnini F Midnight salivary cortisol versus urinary free and midnight serum cortisol as screening tests for Cushing’s syndrome J Clin Endocrinol Metab 2003 88 4153 4157 10.1210/jc.2003-030312 12970280

[B29] Yaneva M Kirilov G Zacharieva S Midnight salivary cortisol, measured by highly sensitive electrochemiluminescence immunoassay, for the diagnosis of Cushing’s syndrome Cent Eur J Med 2009 4 59 64 10.2478/s11536-009-0004-y

[B30] Vogeser M Durner J Seliger E Auernhammer C Measurement of late-night salivary cortisol with an automated immunoassay system Clin Chem Lab Med 2006 44 1441 1445 17163820 10.1515/CCLM.2006.244

[B31] Belaya ZE Iljin AV Melnichenko GA Rozhinskaya LY Dragunova NV Dzeranova LK Butrova SA Troshina EA Dedov II Diagnostic performance of late-night salivary cortisol measured by automated electrochemiluminescence immunoassay in obese and overweight patients referred to exclude Cushing's syndrome Endocrine 2012 41 494 500 10.1007/s12020-012-9658-3 22447310

[B32] Deutschbein T Broecker-Preuss M Flitsch J Jaeger A Althoff R Walz MK Mann K Petersenn S Salivary cortisol as a diagnostic tool for Cushing's syndrome and adrenal insufficiency: improved screening by an automatic immunoassay Eur J Endocrinol 2012 166 613 618 10.1530/EJE-11-0945 22214924

[B33] Putignano P Dubini A Toja P Invitti C Bonfanti S Redaelli G Zappulli D Cavagnini F Salivary cortisol measurement in normal-weight, obese and anorexic women: comparison with plasma cortisol Eur J Endocrinol 2001 145 165 171 10.1530/eje.0.1450165 11454512

[B34] Melin EO Thulesius HO Persson BA Affect School for chronic benign pain patients showed improved alexithymia assessments with TAS-20 Biopsychosoc 2010 4 1 10 10.1186/1751-0759-4-1 PMC2892428 20525319

[B35] Mehling WE Wrubel J Daubenmier J Price CJ Kerr CE Silow T Gopisetty V Stewart AL Body awareness: a phenomenological inquiry into the common ground of mind-body therapies Philos Ethics Humanit Med 2011 6 1 6 10.1186/1747-5341-6-1 21473781 PMC3096919

[B36] Lisspers J Nygren A Soderman E Hospital Anxiety and Depression Scale (HAD): some psychometric data for a Swedish sample Acta Psychiatr Scand 1997 96 281 286 10.1111/j.1600-0447.1997.tb10164.x 9350957

[B37] Lavalard E Szymezak J Leroy N Gillery P Evaluation of variant II analyzer equipped with the new 270-2101 NU kit (Bio-Rad) for HbA 1c assay Ann Biol Clin 2009 67 55 65 10.1684/abc.2008.0289 19189886

[B38] Hoelzel W Weykamp C Jeppsson JO Miedema K Barr JR Goodall I Hoshino T John WG Kobold U Little R Mosca A Mauri P Paroni R Susanto F Takei I Thienpont L Umemoto M Wiedmeyer HM IFCC reference system for measurement of hemoglobin A1c in human blood and the national standardization schemes in the United States, Japan, and Sweden: a method-comparison study Clin Chem 2004 50 166 174 10.1373/clinchem.2003.024802 14709644

